# Plant G × Microbial E: Plant Genotype Interaction with Soil Bacterial Community Shapes Rhizosphere Composition During Invasion

**DOI:** 10.1007/s00248-024-02429-5

**Published:** 2024-09-11

**Authors:** Mae Berlow, Miles Mesa, Mikayla Creek, Jesse G. Duarte, Elizabeth Carpenter, Brandon Phinizy, Krikor Andonian, Katrina M. Dlugosch

**Affiliations:** 1 Department of Ecology and Evolutionary Biology, University of Arizona, Tucson, AZ 85721 USA; 2 Department of Ecology and Evolutionary Biology, University of California, Santa Cruz, CA 95064 USA

**Keywords:** Invasive species, Root microbiome, *Centaurea solstitialis*, Yellow starthistle, Grasslands, Amplicon sequencing

## Abstract

It is increasingly recognized that different genetic variants of hosts can uniquely shape their microbiomes. Invasive species often evolve in their introduced ranges, but little is known about the potential for their microbial associations to change during invasion as a result. We asked whether host genotype (G), microbial environment (E), or their interaction (G × E) affected the composition and diversity of host-associated microbiomes in *Centaurea solstitialis* (yellow starthistle), a Eurasian plant that is known to have evolved novel genotypes and phenotypes and to have altered microbial interactions, in its severe invasion of CA, USA. We conducted an experiment in which native and invading plant genotypes were inoculated with native and invaded range soil microbial communities. We used amplicon sequencing to characterize rhizosphere bacteria in both the experiment and the field soils from which they were derived. We found that native and invading plant genotypes accumulated different microbial associations at the family level in each soil community, often counter to differences in family abundance between soil communities. Root associations with potentially beneficial Streptomycetaceae were particularly interesting, as these were more abundant in the invaded range field soil and accumulated on invading genotypes. We also found that bacterial diversity is higher in invaded soils, but that invading genotypes accumulated a lower diversity of bacteria and unique microbial composition in experimental inoculations, relative to native genotypes. Thus variation in microbial associations of invaders was driven by the interaction of plant G and microbial E, and rhizosphere microbial communities appear to change in composition in response to host evolution during invasion.

**Supplementary Information:**

The online version contains supplementary material available at 10.1007/s00248-024-02429-5.

## Introduction

Introduced species that proliferate in their new environments are able to do so as a result of a suite of environmental and biological interactions [1, 2]. Biotic interactions are thought to be a particularly important means by which an introduced species could become invasive, because of opportunities for escape from enemies (i.e., the Enemy Release Hypothesis [3–7]) or gain of mutualisms [8–11] in an introduced environment. Altered interactions with enemies or mutualists not only could have immediate fitness benefits to invaders but also might allow introduced populations to evolve phenotypes that increase invasiveness, in response to increased resource availability from mutualists and/or relaxed selection for defense from enemies [12, 13]. While the potential for invaders to adapt in response to differences in species interactions in this way has received considerable attention (e.g., the Evolution of Increased Competitive Ability Hypothesis [14, 15]), it is also the case that invader evolution for any reason, adaptive or otherwise, might itself alter species interactions. The question of whether altered species interactions arise from a novel environment (“E”) or from invader evolution (i.e., genotype, “G”), or their interaction (“G × E”) is largely unexplored, though the contributions of these different effects would have important consequences for understanding the mechanisms of invasions and predicting further spread (e.g., see [16, 17]).

For invasive plants, a critical source of both enemy and mutualist interactions is the soil microbial community [5, 18–26]. Some invasive plants perform better with soil microbial communities from invaded ranges, which suggests that initial interactions with soil microbes could facilitate invasion [4, 20, 27–30], but see [31]. Invaders could also benefit from differences in plant-soil feedbacks that accumulate over time [32]. Plants experience negative feedbacks when a host-specific pathogen accumulates in the soil and suppresses their growth [33, 34] or positive feedbacks if the same dynamic occurs with a mutualist [35], and invading species have been observed to benefit from enhanced positive or reduced negative feedbacks in soils from their introduced ranges [24, 29, 35–38]. Invaders might also benefit from interactions with a simplified microbial interaction network in a novel environment, allowing invaders more specificity in exploiting a smaller number of mutualists or defending against fewer pathogens [12, 39].

Regardless of whether microbial pathogens or mutualists differ between native and invaded ranges, we know very little about whether invaders that undergo evolutionary change over the course of their invasion interact with soil microbes differently than native genotypes. It is well documented in the fields of plant disease and agriculture that the genotype of a plant can be tightly correlated with the presence or relative abundance of specific microbes in and around their tissues [40–42]. Plant genotypes can control microbial communities through differences in their resulting phenotype, via traits like root and leaf morphology [43], exudate production [44, 45], and interaction between plant phenotype and environment (e.g., root morphology) [46]. Plant genotypes vary in the amount or type of compounds they produce in defense against microbes [47], or as incidental secondary metabolites that nonetheless impact microbial communities associating with their tissues [48]. These types of genetically based phenotypic differences have been shown to control plant association with individual microbial taxa, as well as the plant-associated microbial community makeup [44, 45, 49]. The strong existing evidence that plant genotype affects microbial associations suggests that the evolution of invader genotype could lead to changes in microbial associations, which could then play a role in invader success and proliferation.

Indeed, invaders are well known to experience rapid evolution for a variety of reasons. Introduced populations can adapt to their new environments and the process of colonization [50–52], but they may also experience non-adaptive genetic changes through several mechanisms, including initial and serial founder events, multiple introductions and admixture of previously isolated subpopulations, hybridization with other species, stimulation of transposable element activity, and/or the revealing of cryptic genetic variation in a novel environment [39, 53–57]. Given that microbial associations respond to host genotype (as discussed above), such broad opportunities for genetic change in invaders are likely to create opportunities for altered species interactions, particularly with microbial communities that are sensitive to variation in plant growth and chemistry.

We test whether invader evolution alters microbial interactions in the invasive annual forb yellow starthistle (*Centaurea solstitialis*, L.; Asteraceae). This species is native to Eurasia, introduced to the Americas, Australia, and South Africa, and highly invasive in the grasslands of North and South America [58]. The *C. solstitialis* invasion of CA, USA, is particularly well characterized for its evolution of novel genotypes and phenotypes. Previous work has used genome-wide markers to reconstruct its invasion history and detail genetic divergence among populations [59–61]. This work finds that plant populations invading California are unique genotypes, genetically divergent from populations elsewhere, but derived from a single subpopulation in Spain and Southern France [59]. Levels of genetic diversity in California populations are similar to those in native range populations, indicating the lack of any strong genetic bottlenecks during the divergence of this invasion [59]. The California genotypes are also distinct from their native progenitors in terms of genetically based phenotypes, including having evolved larger seeds, larger plant size (leaf number, leaf area, height, and biomass), higher reproduction (reproductive duration and number of capitula), and greater competitive ability against grasses than native genotypes [59, 61–65]. Most recently, these phenotypes are being genetically mapped to the regions of the genome responsible for these evolved differences in the invaders [66].

Invading *C. solstitialis* plants in California also appear to accumulate more biomass from more favorable (reduced deleterious) interactions with the soil microbial community in the invaded range [30, 67, 68], raising the possibility that the larger and more fecund invading genotypes have evolved to take advantage of release from microbial enemies or gain of mutualists [69]. These experiments tested the performance of *C. solstitialis* plants in soil inoculated with soil microbial communities collected from different parts of the global range but did not test for evolved differences among host plants. Field experiments treating *C. solstitialis* plots with fungicide found that fungi in the invaded range are less favorable for plant performance, which has generated interest in the potential for bacterial communities to play an important role in creating more favorable growth conditions in the invasion [70]. A previous survey of bacteria associated with *C. solstitialis* in the field by Lu-Irving and colleagues [69] found that bacterial diversity, including of known pathogenic groups, was lower on invading plants. The authors hypothesized that the lower diversity of plant-associated bacteria could reflect escape from enemies in the invaded range [69]. Their results suggest that invaders might benefit from altered interactions with the microbial community, but it is not clear to what extent observed microbiome differences are a cause or effect of the evolution of plant invader genotypes.

Here, we identify the roles of both microbial community environment, plant genotype, and their interaction in shaping plant-microbial associations in this system. Based on previous observations of plant microbiome differences between ranges, we predict that soil microbial communities will be the primary source of variation in microbial associations, and we predict that plants (native or invading genotypes) grown with invaded range soil inoculum will accumulate a lower diversity of rhizosphere bacteria. Using the same populations sampled in previous studies of both plant evolution and microbiome variation in this system [59, 62, 69], we sample field soil from native and invaded ranges to identify the bacterial community available for plant interactions. We conduct a factorial greenhouse experiment, in which both native genotypes and invading genotypes of *C. solstitialis* are grown in native and invaded range soil microbial communities, to identify which members of the soil community recruit to roots of the genotype from each range. We identify bacteria using 16S amplicon sequencing and test for associations with genotype and soil source. In contrast to our predictions, we find that the interaction of both plant genotype and microbial environment influences specific microbial associations, and we find that previously observed patterns of lower diversity of bacterial communities on invading plants are not inherent features of the microbial environment as formerly hypothesized, but are instead the product of novel plant genotype interactions in the invasion.

## Methods

### Study System Biology

*Centaurea solstitialis* was introduced accidentally as a contaminant of alfalfa into South America in the 1600 s and then North America in the 1800s [58, 71]. It is an obligately outcrossing diploid annual plant that has four distinct native genetic subpopulations across Eurasia [59, 60, 72, 73]. In both the California invasion and its source subpopulation in Western Europe, *C. solstitialis* is an annual plant that grows as a rosette with a taproot through a mild and wet winter and spring, then bolts and flowers throughout the dry summer [62, 63, 74].

### Soil and Seed Sampling

Field soils used in this study were collected when seeds were being produced on *C. solstitialis* plants, during dry summer conditions in August 2018. Soils came from four invaded sites in CA, USA, and four native sites in southern Spain and France, all of which were locations previously included in other studies of this system, including in the comparisons of plant-associated microbial diversity by Lu-Irving and colleagues [69], plant genetic diversity by Barker and colleagues [59], and plant trait variation by Dlugosch and colleagues [75] (Supporting Information Table S1). At each site, a 30 m linear sampling transect was established through a *C. solstitialis* patch, and a second 30 m sampling transect was established outside of the patch, parallel to the first and separated by ~ 5 m, to capture site variation outside of *C. solstitialis* patches. The distance between transects was chosen to be adjacent but outside of active growth by *C. solstitialis*, noting that microbial communities have been observed to vary more by soil characteristics than geographic distance even at small scales [76, 77]. Samples from both transects were ultimately grouped for analysis by site (see below). A soil sampling point was established every 2 m along each transect. At each sampling point, an 18 mm diameter soil core was used to sample the topmost 5–10 cm of soil from three adjacent cores (< 10 cm apart), which were combined into a single sample for that sampling point, in a sealed plastic bag. This resulted in 15 separate samples per transect (one per sampling point). Larger bulk soil collections (to be used for experimental inoculum) were made near each transect, both inside and outside of *C. solstitialis* patches. For these bulk collections, 1-L plastic bags were filled with soil collected from the top 5–10 cm of soil from a single location. Gloves and tools were sterilized between soil samples by wiping with 70% isopropanol. Soils were stored dry (as collected) at room temperature until their use in the greenhouse experiment. Seeds were collected from the plant closest to each meter along the transect through the *C. solstitialis* plants, for a total of 30 seed collections per site.

Soil samples were cleaned and homogenized for DNA sampling and use as inoculum. Large rocks and organic particles were removed from each sample manually with sterilized forceps in a biosafety cabinet, and the resulting sample was sifted through a sterilized 2-mm sieve. Soil aggregates that did not pass through the sieve were ground gently with a mortar and pestle to break up the aggregates until all soil particles could pass through the 2-mm sieve. The sample was stirred to homogenize, and 250 mg was weighed and collected in a 2-mL microcentrifuge tube for DNA extraction.

### Experimental Inoculations

We used a fully factorial greenhouse experiment to test for the effects of seed source (native vs. invaded range) and soil microbial community source (native vs. invaded range) combinations on rhizosphere bacterial composition and diversity, using seeds and soils from all sites described above. Soil microbial communities were derived from the bulk collections made from outside of *C. solstitialis* patches at each site (Supporting Information Table S1). All seeds were germinated on the surface of sterile soil (50:50 mix of sand and high porosity soil; HP PRO-MIX™, Quebec, CA) in petri dishes, in a greenhouse set to a maximum of 21 °C day and 10 °C night at the University of Arizona (Tucson, AZ, USA) College of Agriculture and Life Sciences greenhouses in February 2019. The germination date of each seed was noted to account for differences in germination timing (see the “Analyses” section). One week after germinating, seedlings were potted in individual Deepots™ (410 mL; D25L from Stuewe & Sons, OR, USA) in sterile soil and inoculated with a surface application of 15 mL of unsterilized (live) soil slurry from soils collected in the same locations, or sterilized soil as a control. Slurries were prepared by adding 150 mL of sterile water to 10 mL of soil, mixing, and filtering through sterile cotton gauze.

Deepots were arranged in five spatial blocks in the greenhouse. Each block included all four combinations of plants from the native or invaded range planted into soil inoculum from the native or invaded range. Within each of these four combinations were up to 16 combinations of plants and soil inoculum from the four source populations within each range. Multiple source populations were included to capture variation within ranges and were grouped as replicates by range for analyses described below. Replication for each plant range × soil inoculum range combination, including all five blocks, ranged from 43 to 72 plants whose rhizosphere communities were successfully sequenced.

Plants were maintained in the greenhouse for 2 months, when they were harvested by block within a single week. Sterile water was provided through daily misting by a reverse osmosis irrigation system (Evolution-RO, Hydro-Logic Inc., Port Washington, NY, USA). After 1 month, plants were fertilized every 2 weeks using autoclaved Hoagland’s solution (1/8 strength Hoagland Complete Medium, BioWorld, Dublin, OH, USA). At harvest, plants were removed from their pots for rhizosphere collection. The upper 2 to 5 cm of the taproot was collected, together with attached lateral roots, and these root tissues were processed and analyzed together. Excess soil was brushed or shaken off, and root samples were placed in individual 50-mL tubes containing 25 mL of sterile wash solution (45.9 mM NaH_2_PO_4_, 61.6 mM Na_2_HPO_4_, 0.1% Tween 20). Tubes were shaken by hand for 1 min. Root samples were then removed and stored on ice in tubes containing 10 mL of wash solution until further processing, stored on ice during transport, and then refrigerated at 4 °C. Ectorhizosphere washes were centrifuged at 2200 g at 4 °C for 15 min, supernatants were discarded, and pellets were air-dried and stored at 20 °C until DNA extraction. Plant growth and reproduction traits were not collected in this study (but have been extensively studied elsewhere, as described in the “Introduction” section), due to the early age of plants at destructive harvest.

### DNA Extraction and Amplicon Sequencing

Microbial DNA was extracted from soil samples using the DNeasy PowerSoil Pro kit (Qiagen, Hilden, Germany). A blank sample of nanopure water was also included in extractions to record any contamination. The resulting double-stranded DNA concentration was quantified using a Qubit fluorometer (Broad Range Kit, Invitrogen, Waltham, MA, USA).

The 16 s rRNA region was amplified using a 2-step polymerase chain reaction (PCR) as in [69] as follows. Target-specific PCR (PCR 1) was conducted by creating a 25.1 µL reaction mixture using 1 µL of microbial DNA, 1.3 µL of 515-F primer (5′-GTGCCAGCMGCCGCGGTAA-3′) and 1.3 µL of 806-R primer (5′-GGACTACHVGGGTTCTAAT-3′), 12.5 µL of Phusion Flash Master Mix (Thermo Scientific, Waltham, MA, USA), and 9 µL PCR grade water. Reaction mixtures were placed in an Eppendorf Mastercycler Thermal Cycler starting with 98 °C for 10 s, then 25 cycles of this sequence: 98 °C for 1 s, 78 °C for 5 s, 57 °C for 5 s, 72 °C for 15 s, 72 °C for 1 min [78]. PCR 1 products were visualized on an agarose gel to determine whether PCR was successful before a second PCR step (PCR 2) was used to incorporate sequencing adapters onto the ends of the amplified PCR products. PCR 2 reaction mixtures were created using 1 µL of PCR 1 product, 12.5 µL Phusion Flash Master Mix, and 0.75 µL of a unique barcoded primer combination provided by the University of Idaho’s IBEST Genomic Resources Core. Our PCR program ran at 98 °C for 10 s, then 10 cycles of 98 °C for 1 s, 78 °C for 5 s, 51 °C for 5 s, 72 °C for 15 s, 72 °C for 1 min. Barcoded amplicons were quantified by Qubit fluorometry, pooled in equimolar amounts, cleaned using a MinElute kit (Qiagen, Hilden, Germany), and submitted to the University of Idaho’s IBEST Genomic Resources Core for quality control and sequencing with Illumina MiSeq 350 bp pair-end sequencing.

### Analyses

Microbial metabarcoding data was processed and analyzed in QIIME 2 version 2019.10 [79], and additional analyses were carried out in R [80], as detailed below. Scripts for processing sequences and replicating all analyses are available on GitHub (https://github.com/mBerlow/PlantGxMicrobialE.git). Sequences were denoised in QIIME 2 using the Divisive Amplicon Denoising Algorithm (DADA2) to remove sequence errors and trim primers [81]. Next, sequences were aligned, and a phylogeny was generated using FastTree, rooted at the midpoint [82]. Sequences were grouped at the level of amplicon sequence variants (ASVs, 100% similarity), and taxonomy was assigned using the SILVA database, version 138 [83]. ASVs assigned to non-bacterial kingdoms were filtered out for the purposes of our analyses.

Bacterial alpha diversity was measured as richness calculated by the R package “vegetarian” [84] and Shannon’s diversity index calculated by QIIME 2 after rarefying to 1000 sequences per sample [78]. Rarefaction curves indicated that our sequencing coverage was sufficiently exhaustive to observe plateaus in the accumulation of diversity within nearly all of our samples, with a diminishing accumulation of diversity at 1000 sequences (Fig. S1) [79]. To test for the effects of soil source (native vs. invaded range) on alpha diversity metrics, we used two-sided *t*-tests in the stats package in R [80]. To test for effects of experimental treatments on alpha diversity metrics, we used linear models that included effects of seed genotype (native vs. invader), soil source (native vs. invaded range), germination date of the plant (i.e., its age at harvest), experimental block in the greenhouse, and the interaction between seed genotype and soil source (see Figs. S4 & S5 for normality test results). Non-significant interaction terms (*P* > 0.1) were not included in the final models. Linear models and Tukey’s post hoc tests were conducted using the stats package in R [80]. To determine which bacterial taxa were differentially abundant between sample types and treatment groups, we used Linear discriminant analysis Effect Size (LEfSe) [85]. LEfSe identifies taxa whose abundances differ significantly between treatments using a Kruskal Wallace sum-rank test, investigates biological significance with a Wilcoxon rank-sum test, and finally calculates effect sizes of each differentially abundant taxa using linear discriminant analysis (LDA).

Bacterial community beta diversity was measured as unweighted and weighted UniFrac distances, a dissimilarity measure that accounts for phylogenetic relatedness [86]. Unweighted UniFrac distance accounts for information about the presence/absence of ASVs and can be thought of as community membership, while weighted UniFrac distance also accounts for relative abundances and can be thought of as community structure. We used PERMANOVA conducted in the R package vegan [87] with 9999 permutations to test whether beta diversity distances were predicted by fixed effects of range (native vs. invaded) and sites nested within range (including samples both inside and outside of *C. solstitialis* patches). We calculated the partial omega squared effect size using the MicEco package in R.

Given that soil microbial communities can change over very small spatial scales [77], we assessed whether the bulk soil collections (used as inocula for the greenhouse experiment) were representative of the soil bacterial diversity sampled across the transects at the same site. To do this, we compared beta diversity distances between each bulk soil sample and the soil transect samples from the same site with the beta diversity distances between that same bulk soil sample and all the soil samples from other sites. Comparisons were made using paired *t*-tests in R.

## Results

We sequenced a total of 351 samples, yielding 16,654,396 paired reads (mean = 44,059; SD = 35,066; min = 12; max = 173,097; see Table S2 for sequence and ASV counts for each sample and Fig. S1 for rarefaction curves). Sequences are available on the NCBI Sequence Repository (SUB13812121).

### Field Environment: Soils

**Fig. 1:**
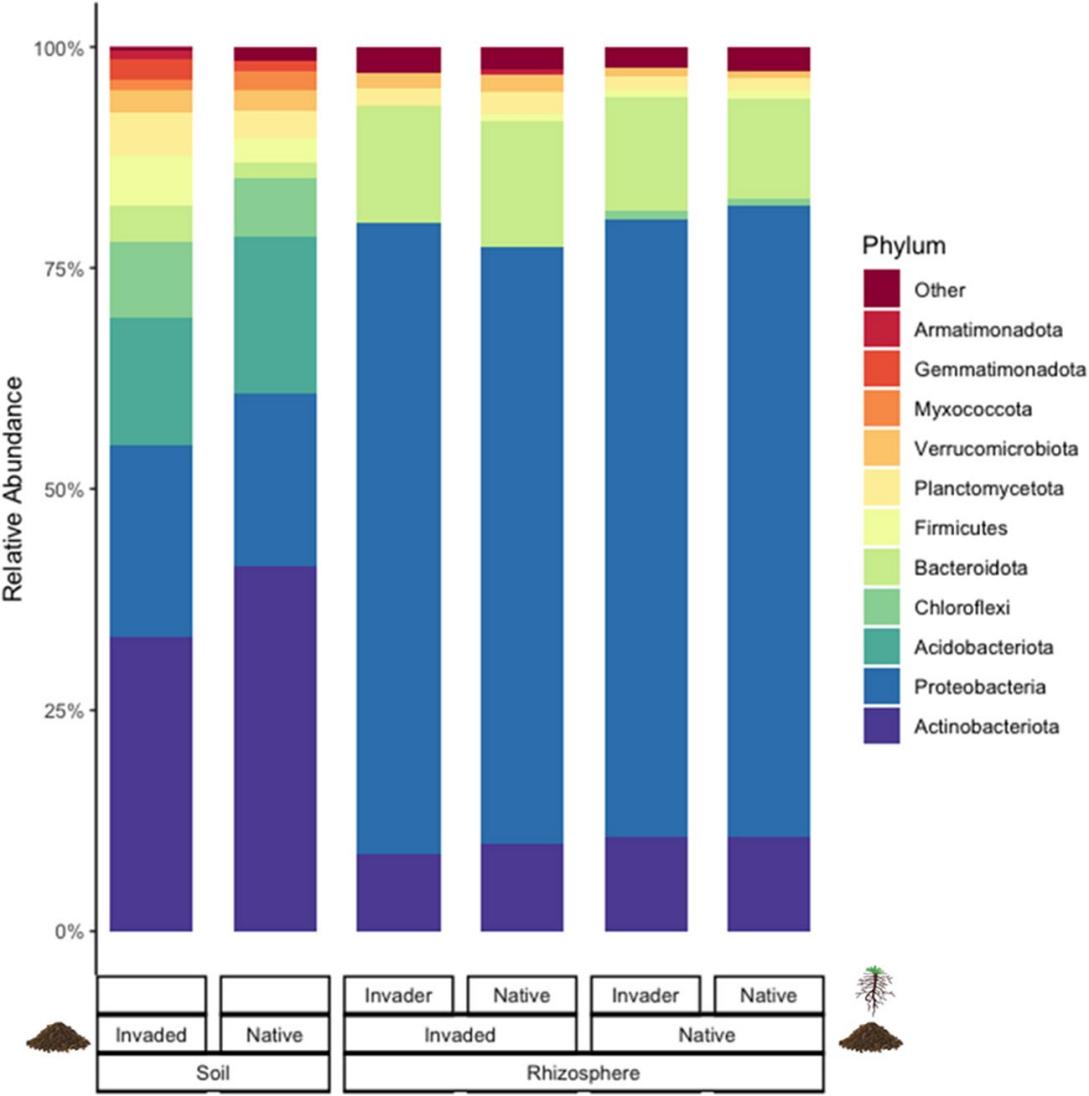
Relative abundance of bacterial phyla in field soil samples, grouped by range (invaded or native) and rhizosphere samples (root wash) grouped by plant genotype (invader or native) within range. All phyla shown constitute at least 1% of at least one sample for soil and at least 0.6% of at least one sample for rhizosphere. The relative abundance of each phylum is average across replicates for each group

Across our native and invaded range field soil samples, we identified 29 phyla, including 294 families of bacteria. Eleven phyla made up 99% of sequences present (Fig. 1, Table S3). The most abundant phyla across all samples were Actinobacteriota (an average of 33% of invaded soil bacterial communities, SD = 9%; 41% of native, SD = 14%), Proteobacteria (22% of invaded, SD = 6%; 20% of native, SD = 14%), and Acidobacteriota (14% of invaded, SD = 5%; 18% of native, SD = 8%).

Bacterial taxa differed between soils from the native and invaded range at both the phylum and family level. Ten phyla were differentially abundant: Actinobacteriota, Acidobacteriota, and Myxococcota were more abundant in native range soils, and Proteobacteria, Armatimonadota, Gemmatimonadota, Planctomycetota, Chloroflexi, Bacteriodota, and Firmicutes were more abundant in invaded range soils (LDA effect size > 2, LEfSe; Fig. S2). Nineteen bacterial families were more abundant in native range soils, and 55 families were more abundant in invaded range soils (LDA effect size > 2, LEfSe; Table S4).

**Fig. 2:**
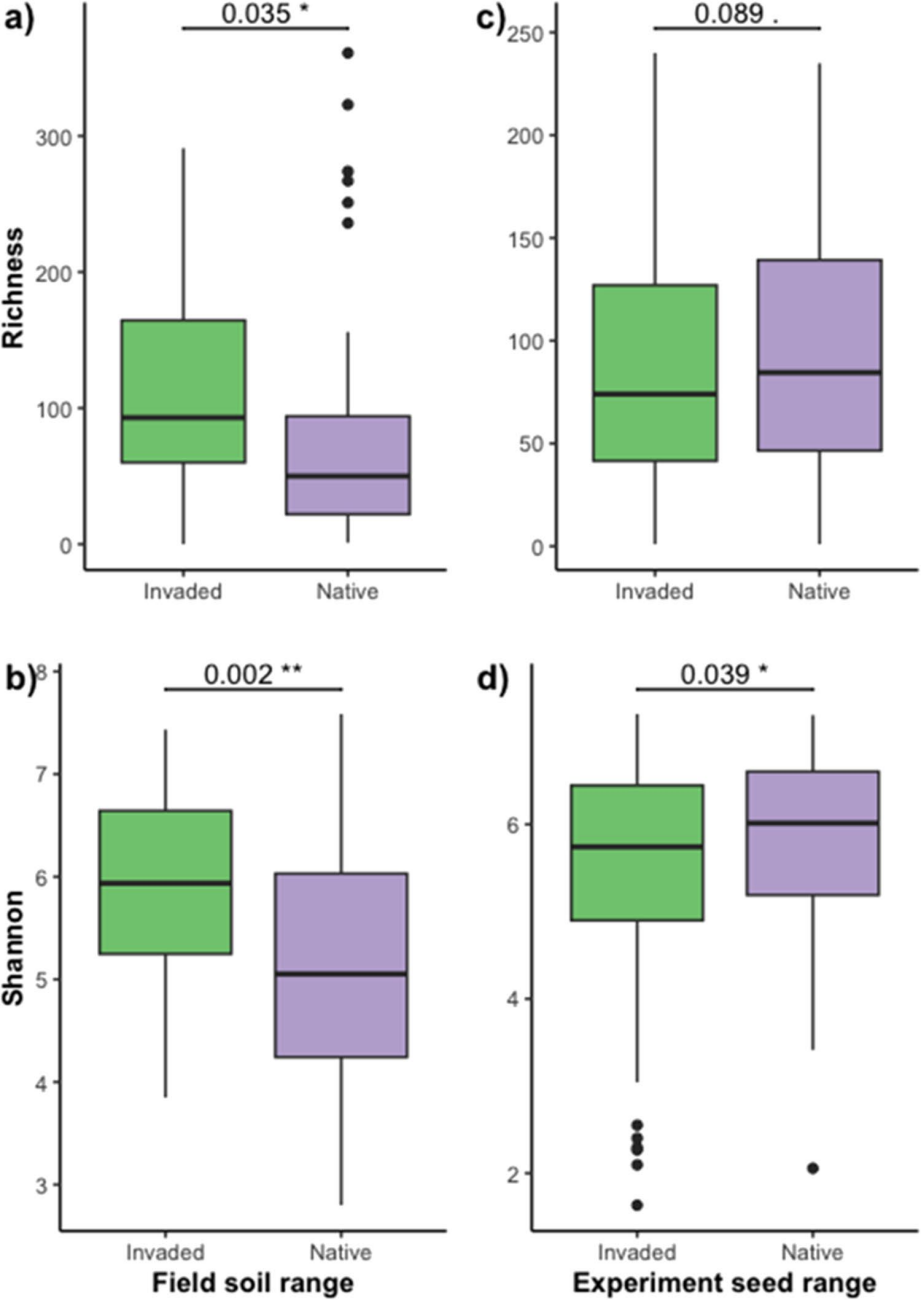
Box plots showing two measures of alpha diversity (**a**, **c** richness; **b**, **d** Shannon’s diversity index) for field soil samples (**a**, **b**) in invaded range soils (CA, USA) and native range soils (Spain and France) and for native and invader yellow starthistle (*C. solstitialis*) genotypes grown (**c**, **d**)

**Fig. 3:**
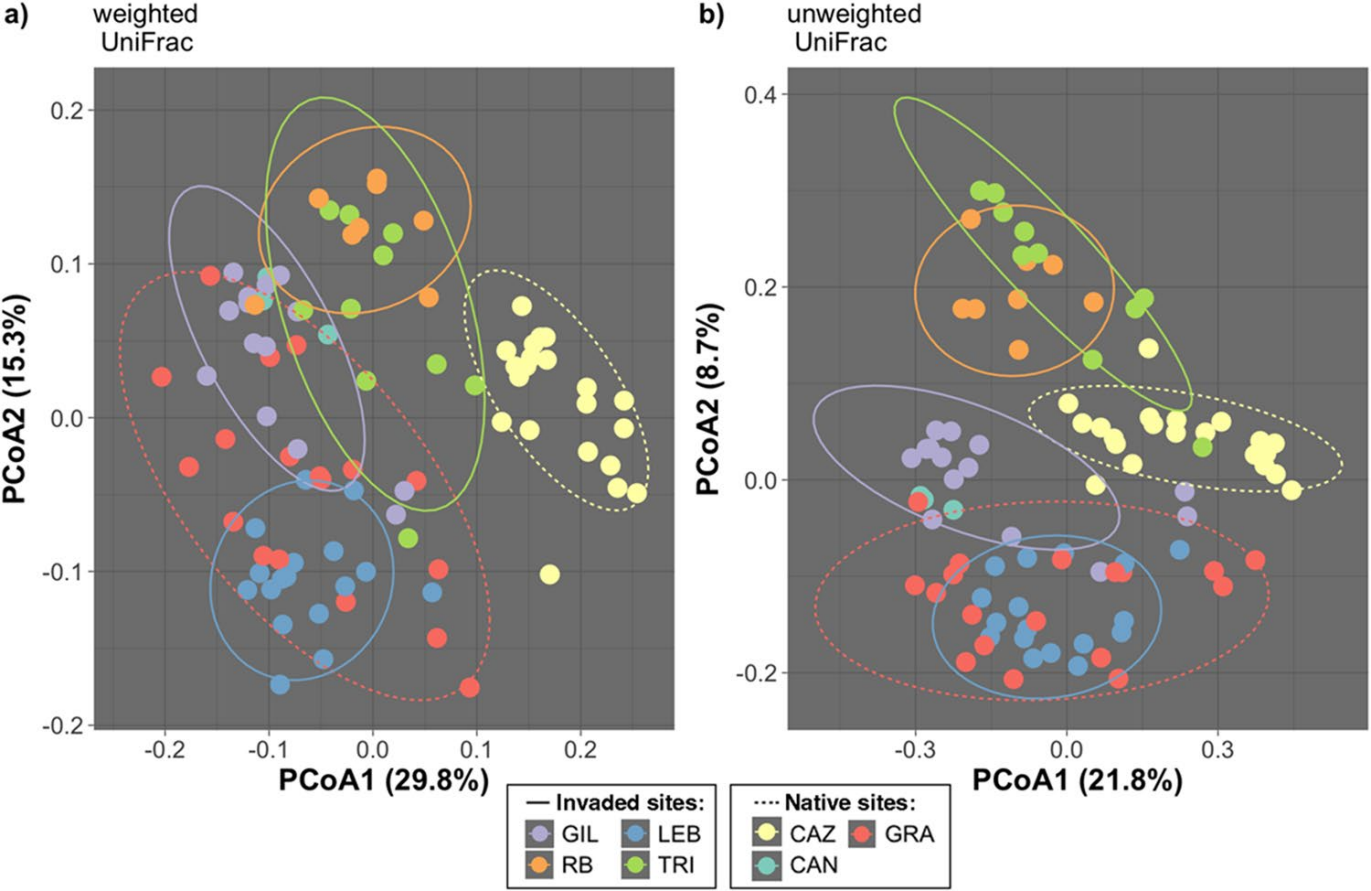
Principal coordinate ordination of **a** weighted UniFrac distances (community structure) and **b** unweighted UniFrac distances (community membership) of field soil samples. Variation explained by each axis is shown in parentheses. Each data point represents one soil sample. Ellipses represent 90% confidence intervals. Solid line ellipses represent invaded sites, and dashed line ellipses represent native sites

The distribution of soil bacterial diversity also differed between the ranges. Invaded soils had higher alpha diversity than native soils by both metrics (richness *P* = 0.035, Shannon *P* = 0.002, *t*-test; Fig. 2a, b). In terms of beta diversity, both range (native or invaded) and site had a significant effect on community membership and community structure (all *P* < 0.0001, PERMANOVA; Table 1; Fig. 3).

**Table 1:** PERMANOVA results for two measures of beta diversity (weighted and unweighted UniFrac)

Predictor	df	Pseudo-*F*	*ω*^2^	*P*
*Unweighted UniFrac:*				
Range	1	5.48	0.06	0.0001
Site with/in range	5	5.49	0.19	0.0001
*Weighted UniFrac:*				
Range	1	10.15	0.14	0.0001
Site with/in range	6	12.51	0.37	0.0001

We found that the distances between the bulk soils used as inocula and the soil samples from the transects at the same site were significantly smaller than the distances between the bulk soils and soil samples from external sites (unweighted UniFrac *P* = 0.005, weighted UniFrac *P* = 0.0001, paired *t*-test, Fig. S3). In other words, bulk soils used as inocula in the greenhouse experiment were more similar to the soil of the site they were from than to any of our other sites and were thus representative of those sites for use in and interpretation of our greenhouse experiments.

### Experimental Inoculation: Rhizosphere

Across our rhizosphere samples, we identified 23 phyla and 352 families of bacteria. Eight phyla made up 99% of sequences present (Fig. 1, Table S5). The most abundant phyla were Proteobacteria (an average of 70% across samples, SD = 12%), Bacteroidota (13%, SD = 9%), and Actinobacteriota (10%, SD = 6%). There were no phyla that differed between native and invading *C. solstitialis* genotypes within either native or invaded soil treatments or between native and invader plant genotypes overall (all LDA effect sizes < 2, LEfSe).

**Fig. 4:**
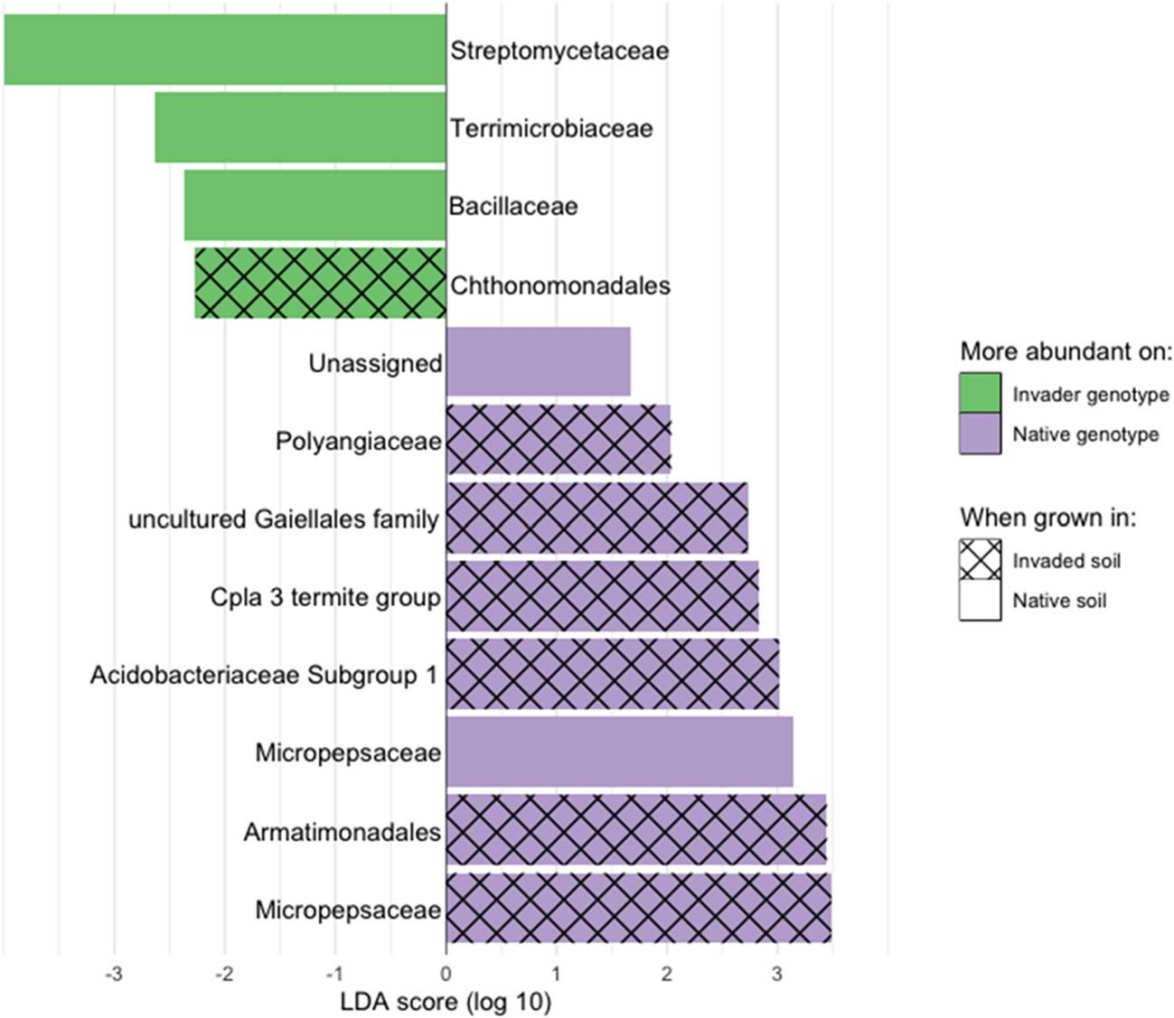
Bacterial families that are differentially abundant in rhizosphere bacterial communities between invader and native *C. solstitialis* genotypes when grown with invaded and native microbial communities. Shown are linear discriminant analysis (LDA) scores from LEfSe analyses comparing rhizosphere microbial communities between native and invading plant genotypes. Solid color bars are from LEfSe comparing microbial communities of each genotype when grown in native soil, hatched bars for when grown in invaded soil. Note: Micropepsaceae was the only family found to be differentially abundant between genotypes in both invaded and native soil

At the bacterial family level, the interaction of the source of soil microbial communities and plant genotype-shaped rhizosphere communities. For invaded range soil inocula, seven bacterial families were differentially abundant, including six families that were more abundant on native range plants and one that was more abundant on invaders (LDA effect size > 2, LEfSe; Fig. 4). For native range soil inocula, five families differed, with two families more abundant on native range plants and three that were more abundant on invaders (LDA effect size > 2, LEfSe; Fig. 4). Only the family Micropepsaceae was differentially abundant between plant genotypes in both native and invaded soil treatments, in which it was more abundant on native genotypes in both treatments.

Contrary to patterns in field soils, alpha diversity (richness and Shannon’s *H*) was higher for bacterial communities on native *C. solstitialis* genotypes than for communities on invader plant genotypes (Fig. 2c, d; Table S6). There was not a significant difference in alpha diversity between communities derived from native vs. invaded range soils, nor an interaction between soil range and plant genotype (Table S6).

## Discussion

Our goal in this study was to test for the effects of plant evolution and its interaction with the soil microbial environment on plant-microbial associations during invasion. In contrast to previous hypotheses that the invaded environment (i.e., Enemy Release) is responsible for shaping microbiome associations for *C. solstitialis* genotypes invading California, we found that both plant genotype and microbial environment interacted to shape microbial family associations in this invasion. We also found that previously observed patterns of plant-associated microbial diversity in this invasion [69] were not a product of microbial communities available in the environment as hypothesized. Instead, plant genotype drove the pattern of its microbiome diversity, such that invading plant genotypes accumulated a lower diversity of rhizosphere bacteria than native genotypes, despite invaded range soils being overall higher in microbial diversity.

Amplicon sequencing of field soils indicated that bacterial community environments differed significantly between ranges and among sites within ranges. Differences between native and invaded microbial environments were evident at both the phylum and family levels. For both taxonomic levels, most differentially abundant taxa were higher in the invaded range soils than the native range soils (e.g., 55 families were more abundant in the invaded range, versus 19 that were elevated in the native range). Consistent with these differences, alpha diversity was higher in the invaded range soils for Shannon diversity and marginally higher for species richness. Soil microbial diversity and composition are known to vary geographically for a variety of reasons, including soil physical characteristics (particularly pH), climatic factors, land use and disturbance, and plant species composition [76, 88, 89]. Our collection sites were typically roadsides adjacent to agricultural fields, with cover of *C. solstitialis* and European grasses, and climatically similar environments [59], in both ranges. Nevertheless, significant side effects within each range indicate that microbial communities were sensitive to subtleties of site characteristics, and the distribution of some microbial taxa (see below) suggests that native range sites might experience more drought stress.

Using these same soils as inocula in our experiment, we found that invading genotypes associated with a lower diversity of bacteria than did native genotypes, regardless of the source of the soil inoculum. This indicates that plant genotypes in this system shape the microbial communities on their roots and that invaders are not only experiencing novel interactions, their evolution is shaping these interactions. In their observational study of *C. solstitialis*–associated microbial communities in the field, Lu-Irving and colleagues (2019) noted that rhizosphere microbial alpha diversity was positively correlated with the genetic diversity (genetic variation) among plants with a site, within each range, consistent with an important effect of different genotypes accumulating a diversity of different microbial taxa within field site. This relationship could not explain differences in microbial diversity between ranges, however, because plant genetic diversity within sites did not differ substantially between the native and invaded ranges in this system [59, 69]. Our experiment reveals that it is a divergence between native and invading plant genotypes, rather than differences in their population genetic diversity within sites, that explains lower rhizosphere diversity on plants in the invasion.

We found no effect of soil community environment on alpha diversity of root-associated microbes in our experiment, but we did find effects of both soil environment and plant genotype on the abundance of specific taxa in the rhizosphere. Below, we discuss known functions of several of these taxa, but we emphasize that their relationships to *C. solstitialis* are unknown in all cases and must be investigated further. While it is difficult to generalize the functions of many bacterial taxa (especially at higher taxonomic ranks) and some are entirely unknown, current understanding of these groups suggests that additional research on their functional roles in this system would be informative.

In possibly our most important finding regarding differentially abundant taxa, we found that Streptomycetaceae were more abundant in invaded range field soils and were more abundant on invader genotypes when grown in the native range soils (in which this group was less abundant, suggesting preferential accumulation). This association between Streptomycetaceae and the invaded range, and the apparent accumulation of these bacteria on invading genotypes, is potentially important because the Streptomycetaceae are widely associated with the promotion of plant growth [90]. Their benefits appear to be both direct effects on growth (as “biofertilizers”) and indirect effects through their suppression of plant pathogens (the Streptomycetaceae are also responsible for the majority of human antibiotics) [91]. If members of this family have a similar positive relationship with invading *C. solstitialis*, this could explain several observed advantages to invaders in this system, including increased invader growth [62], reduced negative plant-soil interactions in the invaded range [30], and potentially the decrease in diversity of microbes on invader roots, given the antimicrobial properties of Streptomycetaceae [69].

Native genotypes also had a set of interactions that mirrored the invader relationships with Streptomycetaceae. These were taxa that were more abundant in native soils and also more abundant on native genotypes when grown in the invaded soils in which these same groups were more depauperate (again suggesting preferential accumulation). These groups have a variety of potential functions of interest and include an uncultured Gaiellales family (a group that has been associated with plant colonizers and may regulate organic and fatty acids in soils [92, 93]), a family in subgroup 1 of the Acidobacteriaceae (a diverse group of oligotrophs abundant in soils, some of which have adapted to specific dry soil conditions; [94, 95]), a family in the CPla-3 termite group (a more highly specialized group in the soil, often in acidic environments; [96]), and the family Polyangiaceae (a group of predatory social bacteria, among the most common predators of bacteria in the soil, with the potential to structure the diversity of the entire microbial community [97–99]).

The most notable association for native plant genotypes, however, appeared to be with the family Micropepsaceae, which was more abundant in invaded range soils, but was overrepresented on native genotypes grown in either invaded or native range soils. This was the only family to be overrepresented on a genotype across both soil environments. The Micropepsaceae has been found to be an indicator taxon associated with specific plant species or site conditions in other studies [100, 101]. For studies that compared different environmental manipulations, the family was associated with early phases of restoration (grasslands [102]), early responses to agricultural planting (tobacco [103]), and response to cold treatments of plants (lettuce [104]), all conditions in which a system had been perturbed recently. In one study, the presence of Micropepsaceae was also associated with reduced suppression of pathogens [105]. If native genotypes are exposed to more pathogens when associating with this family (perhaps during resource acquisition after disturbance), this might favor the diversion of resources from growth info defenses, which has been hypothesized to explain the smaller size of native plant genotypes within *C. solstitialis* [69].

The association patterns of the two families suggest that the native environment might be more stressful for plants, particularly in terms of drought. While native and invaded range climatic niches are generally similar in these areas [59], the families Bacillaceae and Terrimicrobiaceae were more abundant in native range soils. Bacillaceae is characterized by the ability to form endospores that are resistant to environmental extremes, including drought [106]. The Terrimicrobiaceae have been observed to be enriched for G3P (glycerol-3-phosphate) transport-related genes, which are associated with plant–microbe interactions that promote drought tolerance of plants [107]. Both families were also more abundant on invader genotypes in native soils, which is notable because previous research has found that invader genotypes are less drought tolerant and has suggested that this might be adaptive because invaders occupy an environment with lower competition for limiting resources, primarily summer water, and therefore experience lower water stress in the invasion [62].

Finally, two groups that are more common in invaded range soils could be of interest for their differential associations with each genotype growing in that soil inoculum. Armatimonadales is a group of putatively generalist consumers of organic compounds [96, 108]. In a comparison of patches invaded by *Amaranthus palmeri* to patches of native plants in China, Armatimonadales were one of a small number of families strongly associated with the invader and predictive of soil functional characteristics [109]. Taxa in the Chthonomonadales (a group associated with the decomposition of glycine substrates; [110]) were more abundant on invader genotypes in our study. Glycine-rich proteins can be a major component of plant cell walls [111], and associated decomposers may reflect greater nutrient cycling in the presence of higher biomass input [110].

Further work is needed to identify how *C. solstitialis* interactions with these specific bacterial families that we have identified, as well as other components of their microbial communities (i.e., fungi and viruses), affect host plant fitness across ranges. Microbial interactions could be responsible for natural selection that has driven the evolution of invaders in this system, given that root microbiomes can have large effects on host plant performance [112, 113]. A study of 15 annual plant species in California grasslands (not including *C. solstitialis*) found that soil microbial communities generated large fitness differences among species, suggesting that microbially driven selection could be strong [114].

Invaders will also evolve to adapt to other aspects of their new environments and are expected to evolve increases in traits associated with invasiveness itself [50–52, 62, 115–118]. Such trait changes could be synergistic with more favorable microbial interactions, for example, the evolution of increased root investments for resource acquisition could also help plants take advantage of beneficial microbes [119]. The influence of host evolution on microbial interactions during invasion does not appear to have been investigated previously, however. There is some evidence that plant-microbial interactions have changed over time during invasion, which could result from plant evolutionary change. For example, older invading populations of *Solidago canadensis* were found to have increased positive microbial interactions and competitive ability in a common garden experiment, which suggests that these changes were genetically based [120]. In contrast, the evolution of aboveground herbivore interactions in plant invasions has attracted long-standing interest [12], and recent reviews find abundant evidence for the evolution of these interactions, though not consistently to the advantage or disadvantage of the invader [121, 122]. Our work demonstrates that belowground microbial interactions should also be expected to evolve during invasion, as a product of both the resident microbial community and the genetic composition of the host. How these interactions alter fitness and invasiveness over time, and how they interact with available genetic variation in introduced populations, will be important avenues of further research.

## Supplementary Information

Below is the link to the electronic supplementary material.

Supplementary file1 (DOCX 971 KB)

Supplementary file2 (XLSX 126 KB)

## Data Availability

Sequences used in this study are available on the National Center for Biotechnology Information (NCBI) Sequence Repository (SUB13812121). Scripts for processing sequences and replicating all analyses are available on GitHub (https://github.com/mBerlow/PlantGxMicrobialE.git).

## References

[1] Sakai AK, Allendorf FW, Holt JS et al (2001) The population biology of invasive species. Annu Rev Ecol Syst 32:305–332 10.1146/annurev.ecolsys.32.081501.114037

[2] Pearson DE, Ortega YK, Eren Ö, Hierro JL (2018) Community assembly theory as a framework for biological invasions. Trends Ecol Evol 33:313–325 29605085 10.1016/j.tree.2018.03.002

[3] Keane RM, Crawley MJ (2002) Exotic plant invasions and the enemy release hypothesis. Trends Ecol Evol 17:164–170 10.1016/S0169-5347(02)02499-0

[4] Engelkes T, Morriën E, Verhoeven KJF et al (2008) Successful range-expanding plants experience less above-ground and below-ground enemy impact. Nature 456:946–948 19020504 10.1038/nature07474

[5] Colautti RI, Ricciardi A, Grigorovich IA, MacIsaac HJ (2004) Is invasion success explained by the enemy release hypothesis? Ecol Lett 7:721–733 10.1111/j.1461-0248.2004.00616.x

[6] Franks SJ, Pratt PD, Dray FA, Simms EL (2008) Selection on herbivory resistance and growth rate in an invasive plant. Am Nat 171:678–691 18419574 10.1086/587078

[7] Mitchell CE, Blumenthal D, Jarošík V et al (2010) Controls on pathogen species richness in plants’ introduced and native ranges: roles of residence time, range size and host traits. Ecol Lett 13:1525–1535 20973907 10.1111/j.1461-0248.2010.01543.x PMC3003901

[8] Reinhart KO, Callaway RM (2006) Soil biota and invasive plants. New Phytol 170:445–457 16626467 10.1111/j.1469-8137.2006.01715.x

[9] Toby Kiers E, Palmer TM, Ives AR et al (2010) Mutualisms in a changing world: an evolutionary perspective. Ecol Lett 13:1459–1474 20955506 10.1111/j.1461-0248.2010.01538.x

[10] van Kleunen M, Bossdorf O, Dawson W (2018) The ecology and evolution of alien plants. Annu Rev Ecol Evol Syst 49:25–47 10.1146/annurev-ecolsys-110617-062654

[11] Sheng M, Rosche C, Al-Gharaibeh M et al (2022) Acquisition and evolution of enhanced mutualism-an underappreciated mechanism for invasive success? ISME J 16:2467–2478 35871251 10.1038/s41396-022-01293-w PMC9561174

[12] Mesa JM, Dlugosch KM (2020) The evolution of invasiveness: a mechanistic view of trade-offs involving defenses. Am J Bot 107:953–956 10.1002/ajb2.1507

[13] Blumenthal D, Mitchell CE, Pysek P, Jarosík V (2009) Synergy between pathogen release and resource availability in plant invasion. Proc Natl Acad Sci U S A 106:7899–7904 19416888 10.1073/pnas.0812607106 PMC2674393

[14] Blossey B, Notzold R (1995) Evolution of increased competitive ability in invasive nonindigenous plants: a hypothesis. J Ecol 83:887–889 10.2307/2261425

[15] Callaway RM, Lucero JE, Hierro JL, Lortie CJ (2022) The EICA is dead? Long live the EICA! Ecol Lett 25:2289–2302 35986512 10.1111/ele.14088

[16] Huang F, Lankau R, Peng S (2018) Coexistence via coevolution driven by reduced allelochemical effects and increased tolerance to competition between invasive and native plants. New Phytol 218:357–369 29205373 10.1111/nph.14937

[17] Lankau RA, Nuzzo V, Spyreas G, Davis AS (2009) Evolutionary limits ameliorate the negative impact of an invasive plant. Proc Natl Acad Sci 106:15362–15367 19706431 10.1073/pnas.0905446106 PMC2730356

[18] Dawkins K, Mendonca J, Sutherland O, Esiobu N (2022) A systematic review of terrestrial plant invasion mechanisms mediated by microbes and restoration implications. Am J Plant Sci 13:205–222 10.4236/ajps.2022.132013

[19] Rout ME, Callaway RM (2012) Interactions between exotic invasive plants and soil microbes in the rhizosphere suggest that “everything is not everywhere.” Ann Bot 110:213–222 22451600 10.1093/aob/mcs061 PMC3394644

[20] Callaway RM, Thelen GC, Rodriguez A, Holben WE (2004) Soil biota and exotic plant invasion. Nature 427:731–733 14973484 10.1038/nature02322

[21] Torchin ME, Mitchell CE (2004) Parasites, pathogens, and invasions by plants and animals. Front Ecol Environ 2:183–190 10.1890/1540-9295(2004)002[0183:PPAIBP]2.0.CO;2

[22] Agrawal AA, Kotanen PM, Mitchell CE et al (2005) Enemy release? An experiment with congeneric plant pairs and diverse above- and belowground enemies. Ecology 86:2979–2989 10.1890/05-0219

[23] Kulmatiski A, Beard KH, Stevens JR, Cobbold SM (2008) Plant–soil feedbacks: a meta-analytical review. Ecol Lett 11:980–992 18522641 10.1111/j.1461-0248.2008.01209.x

[24] van der Putten WH, Bardgett RD, Bever JD et al (2013) Plant–soil feedbacks: the past, the present and future challenges. J Ecol 101:265–276 10.1111/1365-2745.12054

[25] Faillace CA, Lorusso NS, Duffy S (2017) Overlooking the smallest matter: viruses impact biological invasions. Ecol Lett EarlyView 20:4. 10.1111/ele.12742 10.1111/ele.12742 28176452

[26] Dawson W, Schrama M (2016) Identifying the role of soil microbes in plant invasions. J Ecol 104:1211–1218 10.1111/1365-2745.12619

[27] Reinhart KO, Packer A, Van der Putten WH, Clay K (2003) Plant-soil biota interactions and spatial distribution of black cherry in its native and invasive ranges. Ecol Lett 6:1046–1050 10.1046/j.1461-0248.2003.00539.x

[28] Mitchell CE, Agrawal AA, Bever JD et al (2006) Biotic interactions and plant invasions. Ecol Lett 9:726–740 16706916 10.1111/j.1461-0248.2006.00908.x

[29] Maron JL, Klironomos J, Waller L, Callaway RM (2014) Invasive plants escape from suppressive soil biota at regional scales. J Ecol 102:19–27 10.1111/1365-2745.12172

[30] Andonian K, Hierro JL (2011) Species interactions contribute to the success of a global plant invader. Biol Invasions 13:2957–2965 10.1007/s10530-011-9978-x

[31] Shelby N, Duncan RP, Putten WH et al (2016) Plant mutualisms with rhizosphere microbiota in introduced versus native ranges. J Ecol 104:1259–1270 10.1111/1365-2745.12609

[32] Levine JM, Pachepsky E, Kendall BE et al (2006) Plant-soil feedbacks and invasive spread. Ecol Lett 9:1005–1014 16925649 10.1111/j.1461-0248.2006.00949.x

[33] Bever JD (2003) Soil community feedback and the coexistence of competitors: conceptual frameworks and empirical tests. New Phytol 157:465–473 33873396 10.1046/j.1469-8137.2003.00714.x

[34] Bever JD (2002) Negative feedback within a mutualism: host-specific growth of mycorrhizal fungi reduces plant benefit. Proc Biol Sci 269:2595–2601 12573075 10.1098/rspb.2002.2162 PMC1691198

[35] Uddin MN, Asaeda T, Sarkar A et al (2021) Conspecific and heterospecific plant–soil biota interactions of Lonicera japonica in its native and introduced range: implications for invasion success. Plant Ecol 222:1313–1324 10.1007/s11258-021-01180-y

[36] McGinn KJ, van der Putten WH, Hulme PE et al (2018) The influence of residence time and geographic extent on the strength of plant-soil feedbacks for naturalised Trifolium. J Ecol 106:207–217 10.1111/1365-2745.12864

[37] Meng Y, Geng X, Zhu P et al (2024) Enhanced mutualism: a promotional effect driven by bacteria during the early invasion of Phytolacca americana. Ecol Appl 34:e2742 36107405 10.1002/eap.2742

[38] Suding KN, Stanley Harpole W, Fukami T et al (2013) Consequences of plant–soil feedbacks in invasion. J Ecol 101:298–308 10.1111/1365-2745.12057

[39] Colautti RI, Lau JA (2015) Contemporary evolution during invasion: evidence for differentiation, natural selection, and local adaptation. Mol Ecol 24:1999–2017 25891044 10.1111/mec.13162

[40] Yue H, Sun X, Wang T et al (2024) Host genotype-specific rhizosphere fungus enhances drought resistance in wheat. Microbiome 12:44 38433268 10.1186/s40168-024-01770-8 PMC10910722

[41] Schweitzer JA, Bailey JK, Fischer DG et al (2008) Plant-soil microorganism interactions: heritable relationship between plant genotype and associated soil microorganisms. Ecology 89:773–781 18459340 10.1890/07-0337.1

[42] Aira M, Gómez-Brandón M, Lazcano C et al (2010) Plant genotype strongly modifies the structure and growth of maize rhizosphere microbial communities. Soil Biol Biochem 42:2276–2281 10.1016/j.soilbio.2010.08.029

[43] King WL, Yates CF, Guo J et al (2021) The hierarchy of root branching order determines bacterial composition, microbial carrying capacity and microbial filtering. Commun Biol 4:483 33875783 10.1038/s42003-021-01988-4 PMC8055976

[44] Saunders M, Kohn LM (2009) Evidence for alteration of fungal endophyte community assembly by host defense compounds. New Phytol 182:229–238 19170900 10.1111/j.1469-8137.2008.02746.x

[45] Bailey JK, Deckert R, Schweitzer JA et al (2005) Host plant genetics affect hidden ecological players: links among Populus, condensed tannins, and fungal endophyte infection. Can J Bot 83:356–361 10.1139/b05-008

[46] Lareen A, Burton F, Schäfer P (2016) Plant root-microbe communication in shaping root microbiomes. Plant Mol Biol 90:575–587 26729479 10.1007/s11103-015-0417-8 PMC4819777

[47] Van der Ent S, Verhagen BWM, Van Doorn R et al (2008) MYB72 is required in early signaling steps of rhizobacteria-induced systemic resistance in Arabidopsis. Plant Physiol 146:1293–1304 18218967 10.1104/pp.107.113829 PMC2259080

[48] Voges MJEEE, Bai Y, Schulze-Lefert P, Sattely ES (2019) Plant-derived coumarins shape the composition of an Arabidopsis synthetic root microbiome. Proc Natl Acad Sci U S A 116:12558–12565 31152139 10.1073/pnas.1820691116 PMC6589675

[49] Ahlholm JU, Helander M, Henriksson J et al (2002) Environmental conditions and host genotype direct genetic diversity of Venturia ditricha, a fungal endophyte of birch trees. Evolution 56:1566–1573 12353749 10.1111/j.0014-3820.2002.tb01468.x

[50] Williams JL, Snyder RE, Levine JM (2016) The influence of evolution on population spread through patchy landscapes. Am Nat 188:15–26 27322118 10.1086/686685

[51] Williams JL, Kendall BE, Levine JM (2016) Rapid evolution accelerates plant population spread in fragmented experimental landscapes. Science 353:482–485 27471303 10.1126/science.aaf6268

[52] Szűcs M, Vahsen ML, Melbourne BA et al (2017) Rapid adaptive evolution in novel environments acts as an architect of population range expansion. Proc Natl Acad Sci U S A 114:13501–13506 29183976 10.1073/pnas.1712934114 PMC5754790

[53] Dlugosch KM, Anderson SR, Braasch J et al (2015) The devil is in the details: genetic variation in introduced populations and its contributions to invasion. Mol Ecol 24:2095–2111 25846825 10.1111/mec.13183

[54] Dlugosch KM, Parker IM (2008) Founding events in species invasions: genetic variation, adaptive evolution, and the role of multiple introductions. Mol Ecol 17:431–449 17908213 10.1111/j.1365-294X.2007.03538.x

[55] Lee CE (2002) Evolutionary genetics of invasive species. Trends Ecol Evol 17:386–391 10.1016/S0169-5347(02)02554-5

[56] Bock DG, Caseys C, Cousens RG et al (2015) What we still don’t know about invasion genetics. Mol Ecol 24:2277–2297 25474505 10.1111/mec.13032

[57] Prentis PJ, Wilson JRU, Dormontt EE et al (2008) Adaptive evolution in invasive species. Trends Plant Sci 13:288–294 18467157 10.1016/j.tplants.2008.03.004

[58] Gerlach JD (1997) How the west was lost: reconstructing the invasion dynamics of yellow starthistle and other plant invaders of western rangelands and natural areas. Calif Exotic Pest Plant Counc Symp Proc 3:67–72

[59] Barker BS, Andonian K, Swope SM et al (2017) Population genomic analyses reveal a history of range expansion and trait evolution across the native and invaded range of yellow starthistle (Centaurea solstitialis). Mol Ecol 26:1131–1147 28029713 10.1111/mec.13998 PMC5480294

[60] Eriksen RL, Hierro JL, Eren Ö et al (2014) Dispersal pathways and genetic differentiation among worldwide populations of the invasive weed Centaurea solstitialis L. (Asteraceae). PLoS One 9:e114786 25551223 10.1371/journal.pone.0114786 PMC4281129

[61] Irimia RE, Montesinos D, Chaturvedi A et al (2023) Trait evolution during a rapid global weed invasion despite little genetic differentiation. Evol Appl 16:997–1011 37216028 10.1111/eva.13548 PMC10197227

[62] Dlugosch KM, Alice Cang F, Barker BS et al (2015) Evolution of invasiveness through increased resource use in a vacant niche. Nature Plants 1:15066 26770818 10.1038/nplants.2015.66 PMC4710175

[63] Widmer TL, Guermache F, Dolgovskaia MY, Reznik SY (2007) Enhanced growth and seed properties in introduced vs. native populations of yellow starthistle (Centaurea solstitialis). Weed Sci 55:465–473 10.1614/WS-06-211R.1

[64] Eriksen RL, Desronvil T, Hierro JL, Kesseli R (2012) Morphological differentiation in a common garden experiment among native and non-native specimens of the invasive weed yellow starthistle (Centaurea solstitialis). Biol Invasions 14:1459–1467 10.1007/s10530-012-0172-6

[65] Montesinos D, Graebner RC, Callaway RM (2019) Evidence for evolution of increased competitive ability for invasive Centaurea solstitialis, but not for naturalized C. calcitrapa. Biol Invasions 21:99–110 10.1007/s10530-018-1807-z

[66] Reatini B, Alice Cang F, Jiang Q et al (2022) Chromosome-scale reference genome and RAD-based genetic map of yellow starthistle (Centaurea solstitialis) reveal putative structural variation and QTLs associated with invader traits. bioRxiv preprint 09.28.509992. 10.1101/2022.09.28.509992 10.1093/gbe/evae243 PMC11632367 39592405

[67] Andonian K, Hierro JL, Khetsuriani L et al (2012) Geographic mosaics of plant-soil microbe interactions in a global plant invasion. J Biogeogr 39:600–608 10.1111/j.1365-2699.2011.02629.x

[68] Andonian K, Hierro JL, Khetsuriani L et al (2011) Range-expanding populations of a globally introduced weed experience negative plant-soil feedbacks. PLoS ONE 6:e20117 21629781 10.1371/journal.pone.0020117 PMC3100334

[69] Lu-Irving P, Harenčár JG, Sounart H et al (2019) Native and invading yellow starthistle (Centaurea solstitialis) microbiomes differ in composition and diversity of bacteria. mSphere 4:2. 10.1128/mSphere.00088-19 10.1128/mSphere.00088-19 PMC6403453 30842267

[70] Hierro JL, Khetsuriani L, Andonian K et al (2016) The importance of factors controlling species abundance and distribution varies in native and non-native ranges. Ecography EarlyView 40:8. 10.1111/ecog.02633 10.1111/ecog.02633

[71] DiTomaso JM, Healy EA (2007) Weeds of California and other western states. University of California Department of Agriculture and Natural Resources, Oakland, CA

[72] Sun M (1997) Population genetic structure of yellow starthistle (Centaurea solstitialis), a colonizing weed in the western United States. Can J Bot 75:1470–1478 10.1139/b97-861

[73] Irimia R-E, Montesinos D, Eren Ö et al (2017) Extensive analysis of native and non-native Centaurea solstitialis L. populations across the world shows no traces of polyploidization. PeerJ 5:e3531 28828232 10.7717/peerj.3531 PMC5560225

[74] Hierro JL, Eren Ö, Khetsuriani L et al (2009) Germination responses of an invasive species in native and non-native ranges. Oikos 118:529–538 10.1111/j.1600-0706.2008.17283.x

[75] Dlugosch KM, Cang FA, Barker BS et al (2015) Evolution of invasiveness through increased resource use in a vacant niche. Nat Plants 1:6. 10.1038/nplants.2015.66 10.1038/nplants.2015.66 PMC4710175 26770818

[76] Fierer N, Jackson RB (2006) The diversity and biogeography of soil bacterial communities. Proc Natl Acad Sci U S A 103:626–631 16407148 10.1073/pnas.0507535103 PMC1334650

[77] Pasternak Z, Al-Ashhab A, Gatica J et al (2013) Spatial and temporal biogeography of soil microbial communities in arid and semiarid regions. PLoS ONE 8:e69705 23922779 10.1371/journal.pone.0069705 PMC3724898

[78] Lundberg DS, Yourstone S, Mieczkowski P et al (2013) Practical innovations for high-throughput amplicon sequencing. Nat Methods 10:999–1002 23995388 10.1038/nmeth.2634

[79] Bolyen E, Rideout JR, Dillon MR et al (2019) Reproducible, interactive, scalable and extensible microbiome data science using QIIME 2. Nat Biotechnol 37(8):852–857. 10.1038/s41587-019-0209-9 31341288 10.1038/s41587-019-0209-9 PMC7015180

[80] R Core Team (2019) R: a language and environment for statistical computing. Version 3.6. 0. Vienna, Austria. R Foundation for Statistical Computing, Vienna, Austria. URL https://www.R-Project.org

[81] Rosen MJ, Callahan BJ, Fisher DS, Holmes SP (2012) Denoising PCR-amplified metagenome data. BMC Bioinformatics 13:1–16 23113967 10.1186/1471-2105-13-283 PMC3563472

[82] Price MN, Dehal PS, Arkin AP (2010) FastTree 2–approximately maximum-likelihood trees for large alignments. PLoS ONE 5:e9490 20224823 10.1371/journal.pone.0009490 PMC2835736

[83] Quast C, Pruesse E, Yilmaz P et al (2013) The SILVA ribosomal RNA gene database project: improved data processing and web-based tools. Nucleic Acids Res. 10.1093/nar/gks1219 23193283 10.1093/nar/gks1219 PMC3531112

[84] Jost L (2007) Partitioning diversity into independent alpha and beta components. Ecology 88:2427–2439 18027744 10.1890/06-1736.1

[85] Segata N, Izard J, Waldron L et al (2011) Metagenomic biomarker discovery and explanation. Genome Biol 12:R60 21702898 10.1186/gb-2011-12-6-r60 PMC3218848

[86] Lozupone C, Lladser ME, Knights D et al (2011) UniFrac: an effective distance metric for microbial community comparison. ISME J 5:169–172 20827291 10.1038/ismej.2010.133 PMC3105689

[87] Oksanen J, Blanchet FG, Friendly M, Kindt R, Legendre P, McGlinn D, Minchin PR, O'Hara R B, Simpson GL, Solymos P, Stevens MHH, Szoecs E, Wagner H (2019) Vegan: community ecology package. R package version 2.5-6. https://CRAN.R-project.org/package=vegan

[88] Nottingham AT, Fierer N, Turner BL et al (2018) Microbes follow Humboldt: temperature drives plant and soil microbial diversity patterns from the Amazon to the Andes. Ecology 99:2455–2466 30076592 10.1002/ecy.2482 PMC6850070

[89] Chu H, Gao G-F, Ma Y et al (2020) Soil microbial biogeography in a changing world: recent advances and future perspectives. mSystems 5:2. 10.1128/mSystems.00803-19 10.1128/mSystems.00803-19 PMC7174637 32317392

[90] Viaene T, Langendries S, Beirinckx S et al (2016) Streptomyces as a plant’s best friend? FEMS Microbiol Ecol 92:8. 10.1093/femsec/fiw119 10.1093/femsec/fiw119 27279415

[91] Olanrewaju OS, Babalola OO (2019) Streptomyces: implications and interactions in plant growth promotion. Appl Microbiol Biotechnol 103:1179–1188 30594952 10.1007/s00253-018-09577-y PMC6394478

[92] Ye F, Wang X, Wang Y et al (2021) Different pioneer plant species have similar rhizosphere microbial communities. Plant Soil 464:165–181 10.1007/s11104-021-04952-7

[93] Sun L, Wang Y, Ma D et al (2022) Differential responses of the rhizosphere microbiome structure and soil metabolites in tea (Camellia sinensis) upon application of cow manure. BMC Microbiol 22:55 35164712 10.1186/s12866-022-02470-9 PMC8842532

[94] Kielak AM, Barreto CC, Kowalchuk GA et al (2016) The ecology of Acidobacteria: moving beyond genes and genomes. Front Microbiol 7:744 27303369 10.3389/fmicb.2016.00744 PMC4885859

[95] Sikorski J, Baumgartner V, Birkhofer K et al (2022) The evolution of ecological diversity in Acidobacteria. Front Microbiol 13:715637 35185839 10.3389/fmicb.2022.715637 PMC8847707

[96] Fickling NW, Abbott CA, Brame JE et al (2024) Light-dark cycles may influence in situ soil bacterial networks and diurnally-sensitive taxa. Ecol Evol 14:e11018 38357595 10.1002/ece3.11018 PMC10864733

[97] Petters S, Groß V, Söllinger A et al (2021) The soil microbial food web revisited: predatory myxobacteria as keystone taxa? ISME J 15:2665–2675 33746204 10.1038/s41396-021-00958-2 PMC8397742

[98] Wu Z, Li Y, Chen H et al (2022) Effects of straw mulching on predatory myxobacterial communities in different soil aggregates under wheat-corn rotation. Environ Sci Pollut Res Int 29:29062–29074 34993829 10.1007/s11356-021-18350-0

[99] Thakur MP, Geisen S (2019) Trophic regulations of the soil microbiome. Trends Microbiol 27:771–780 31138481 10.1016/j.tim.2019.04.008

[100] Wu D, Bai H, Zhao C et al (2023) The characteristics of soil microbial co-occurrence networks across a high-latitude forested wetland ecotone in China. Front Microbiol 14:1160683 37025633 10.3389/fmicb.2023.1160683 PMC10072330

[101] Gschwend F, Hartmann M, Mayerhofer J et al (2022) Site and land-use associations of soil bacteria and fungi define core and indicative taxa. FEMS Microbiol Ecol 97:12. 10.1093/femsec/fiab165 10.1093/femsec/fiab165 PMC8752248 34940884

[102] Barber NA, Klimek DM, Bell JK, Swingley WD (2023) Restoration age and reintroduced bison may shape soil bacterial communities in restored tallgrass prairies. FEMS Microbiol Ecol 99:3. 10.1093/femsec/fiad007 10.1093/femsec/fiad007 36669763

[103] Cao Y, Yang Z-X, Yang D-M et al (2022) Tobacco root microbial community composition significantly associated with root-knot nematode infections: dynamic changes in microbiota and growth stage. Front Microbiol 13:807057 35222332 10.3389/fmicb.2022.807057 PMC8863970

[104] Persyn A, Garcia Mendez S, Beirinckx S et al (2022) Digging into the lettuce cold-specific root microbiome in search of chilling stress tolerance-conferring plant growth-promoting bacteria. Phytobiomes J. 10.1094/PBIOMES-07-22-0044-MF 10.1094/PBIOMES-07-22-0044-MF

[105] Yu L, Zi H, Zhu H et al (2022) Rhizosphere microbiome of forest trees is connected to their resistance to soil-borne pathogens. Plant Soil 479:143–158 10.1007/s11104-022-05505-2

[106] Mandic-Mulec I, Stefanic P, van Elsas JD (2016) Ecology of *Bacillaceae*. The bacterial spore. ASM Press, Washington, DC, USA, pp 59–85

[107] Styer A, Pettinga D, Caddell D, Coleman-Derr D (2024) Improving rice drought tolerance through host-mediated microbiome selection. bioRxiv preprint 02.03.578672. 10.1101/2024.02.03.578672

[108] Kato S, Masuda S, Shibata A et al (2022) Insights into ecological roles of uncultivated bacteria in Katase hot spring sediment from long-read metagenomics. Front Microbiol 13:1045931 36406403 10.3389/fmicb.2022.1045931 PMC9671151

[109] Zhang M, Shi C, Li X et al (2023) Changes in the structure and function of rhizosphere soil microbial communities induced by Amaranthus palmeri invasion. Front Microbiol 14:1114388 37056750 10.3389/fmicb.2023.1114388 PMC10089265

[110] Bhatnagar JM, Peay KG, Treseder KK (2018) Litter chemistry influences decomposition through activity of specific microbial functional guilds. Ecol Monogr 88:429–444 10.1002/ecm.1303

[111] Ringli C, Keller B, Ryser U (2001) Glycine-rich proteins as structural components of plant cell walls. Cell Mol Life Sci 58:1430–1441 11693524 10.1007/PL00000786 PMC11337278

[112] Bai B, Liu W, Qiu X et al (2022) The root microbiome: community assembly and its contributions to plant fitness. J Integr Plant Biol 64:230–243 35029016 10.1111/jipb.13226

[113] Trivedi P, Leach JE, Tringe SG et al (2020) Plant-microbiome interactions: from community assembly to plant health. Nat Rev Microbiol 18:607–621 32788714 10.1038/s41579-020-0412-1

[114] Kandlikar G, Yan X, Levine JM, Kraft NJB (2021) Soil microbes generate stronger fitness differences than stabilization among California annual plants. Am Nat 197:E30–E39 33417516 10.1086/711662

[115] Weiss-Lehman C, Hufbauer RA, Melbourne BA (2017) Rapid trait evolution drives increased speed and variance in experimental range expansions. Nat Commun 8:14303 28128350 10.1038/ncomms14303 PMC5290145

[116] Perkins TA, Phillips BL, Baskett ML, Hastings A (2013) Evolution of dispersal and life history interact to drive accelerating spread of an invasive species. Ecol Lett 16:1079–1087 23809102 10.1111/ele.12136

[117] Gioria M, Hulme PE, Richardson DM, Pyšek P (2023) Why are invasive plants successful? Annu Rev Plant Biol 74:635–670 36750415 10.1146/annurev-arplant-070522-071021

[118] McGaughran A, Dhami MK, Parvizi E et al (2024) Genomic tools in biological invasions: current state and future frontiers. Genome Biol Evol 16:1. 10.1093/gbe/evad230 10.1093/gbe/evad230 PMC10776249 38109935

[119] Dawson W (2015) Release from belowground enemies and shifts in root traits as interrelated drivers of alien plant invasion success: a hypothesis. Ecol Evol 5:4505–4516 26668717 10.1002/ece3.1725 PMC4670063

[120] Oduor AMO, Adomako MO, Yuan Y, Li J-M (2022) Older populations of the invader Solidago canadensis exhibit stronger positive plant-soil feedbacks and competitive ability in China. Am J Bot 109:1230–1241 35819013 10.1002/ajb2.16034

[121] Gruntman M, Segev U (2024) Effect of residence time on trait evolution in invasive plants: review and meta-analysis. NBER Work Pap Ser 91:99–124

[122] Yi J, Wan J, Tielbörger K et al (2024) Specialist reassociation and residence time modulate the evolution of defense in invasive plants: a meta-analysis. Ecology 105:e4253 38272490 10.1002/ecy.4253

