# Supplementary material for: Plant G × Microbial E: Plant Genotype Interaction with Soil Bacterial Community Shapes Rhizosphere Composition During Invasion
Source: Microb Ecol. 2024 Sep 11;87(1):113. doi: 10.1007/s00248-024-02429-5 (PMC11390927; doi:10.1007/s00248-024-02429-5)
Supplement: Supplementary file 1 — Supplementary file1 (DOCX 971 KB) [file 248_2024_2429_MOESM1_ESM.docx]

Supporting Figures for “Plant G x Microbial E: Plant genotype interaction with soil bacterial community shapes rhizosphere composition during invasion”


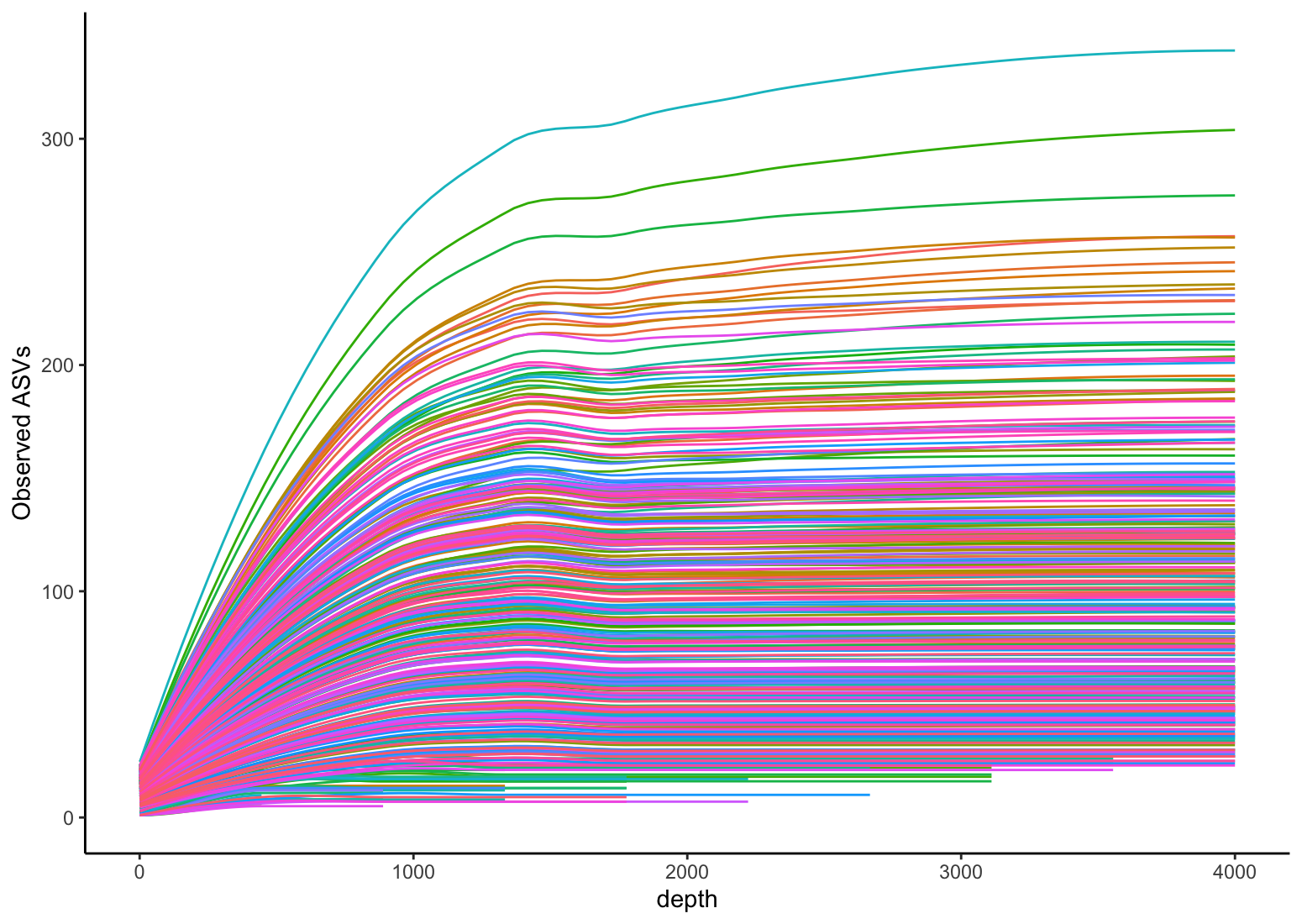


Figure S1 – Rarefaction curve showing that amplicon sequence variant (ASV) accumulation reached a plateau for most samples, indicating that sequencing had sufficient depth to capture bacterial diversity of samples. A rarefaction depth of 1000 maximized observed ASVs while preserving most samples.


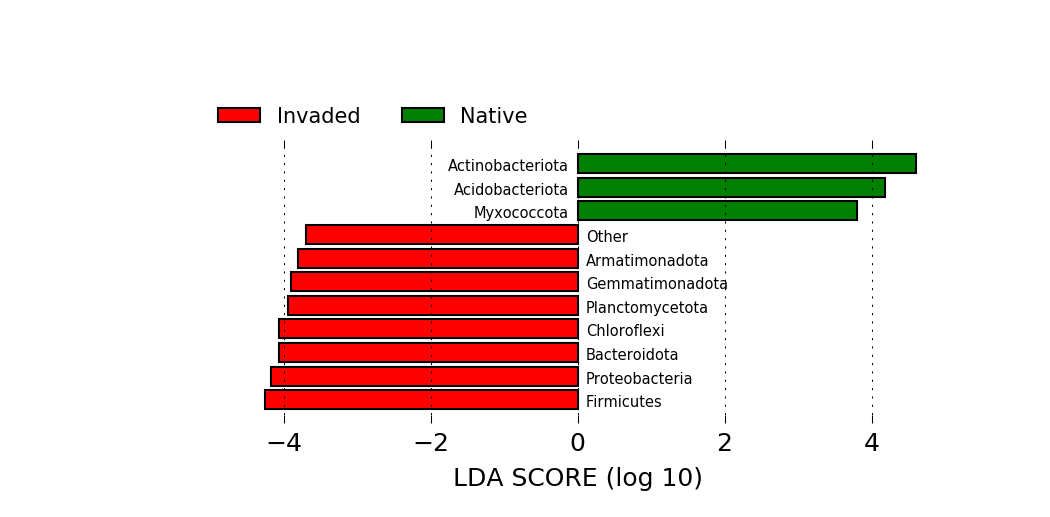


Figure S2 - Bacterial phyla that are differentially abundant in soil bacterial communities between invaded and native ranges of *C. solstitialis*. Results from linear discriminant analysis effect size (LEfSe).


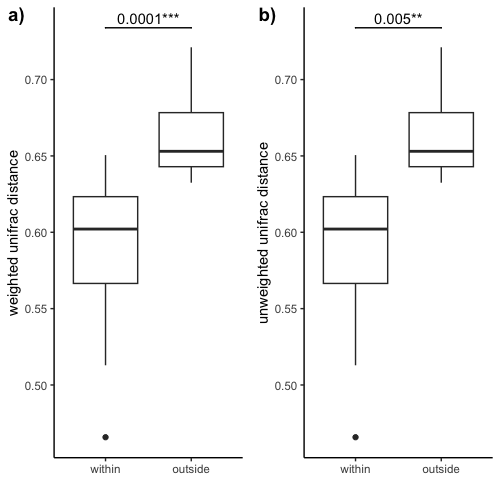


Figure S3 - Comparison of bulk soil samples used as inocula in greenhouse experiment compared to other soil samples **within** the same site, and **outside** the site they were collected from. Bulk soils used as inocula in the greenhouse experiment were more similar to the soil of the site they were from than to any of our other sites, and were thus representative of those sites for use in and interpretation of our greenhouse experiments.


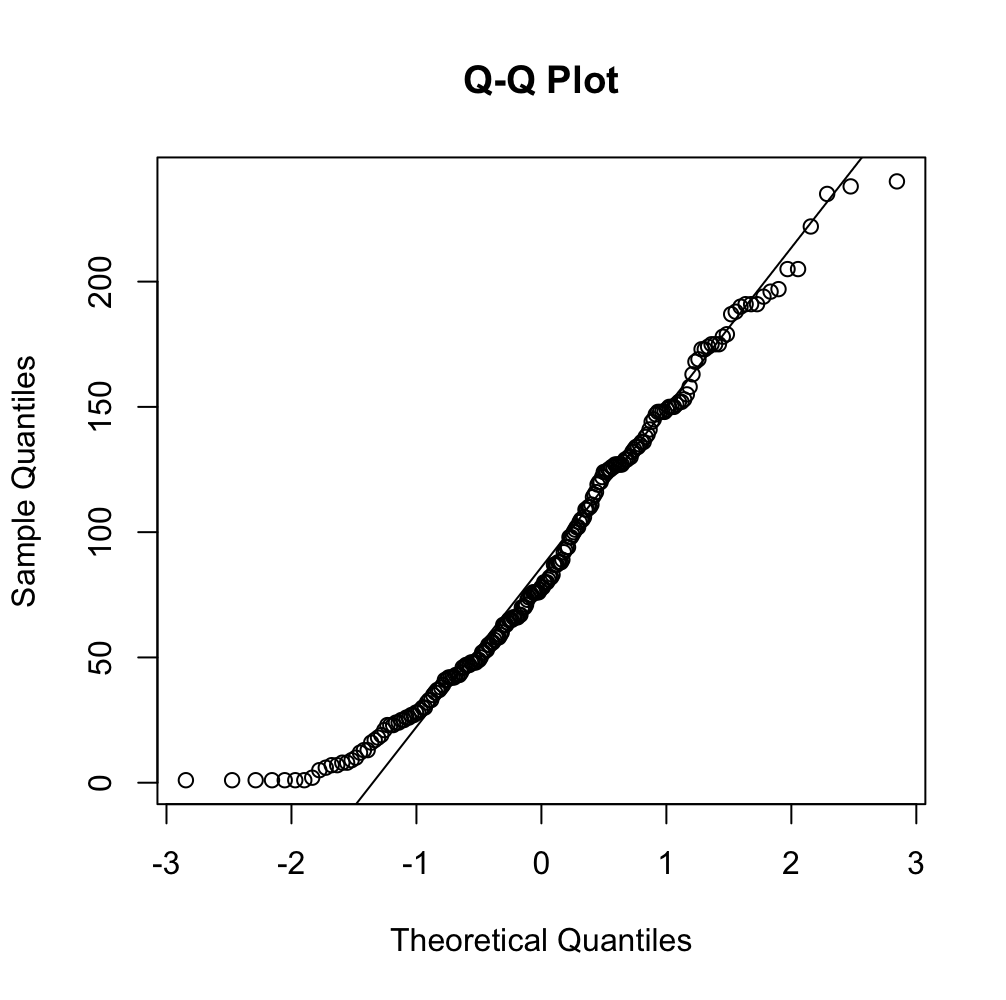


Figure S4 - Q-Q plot of Shannon Index data.


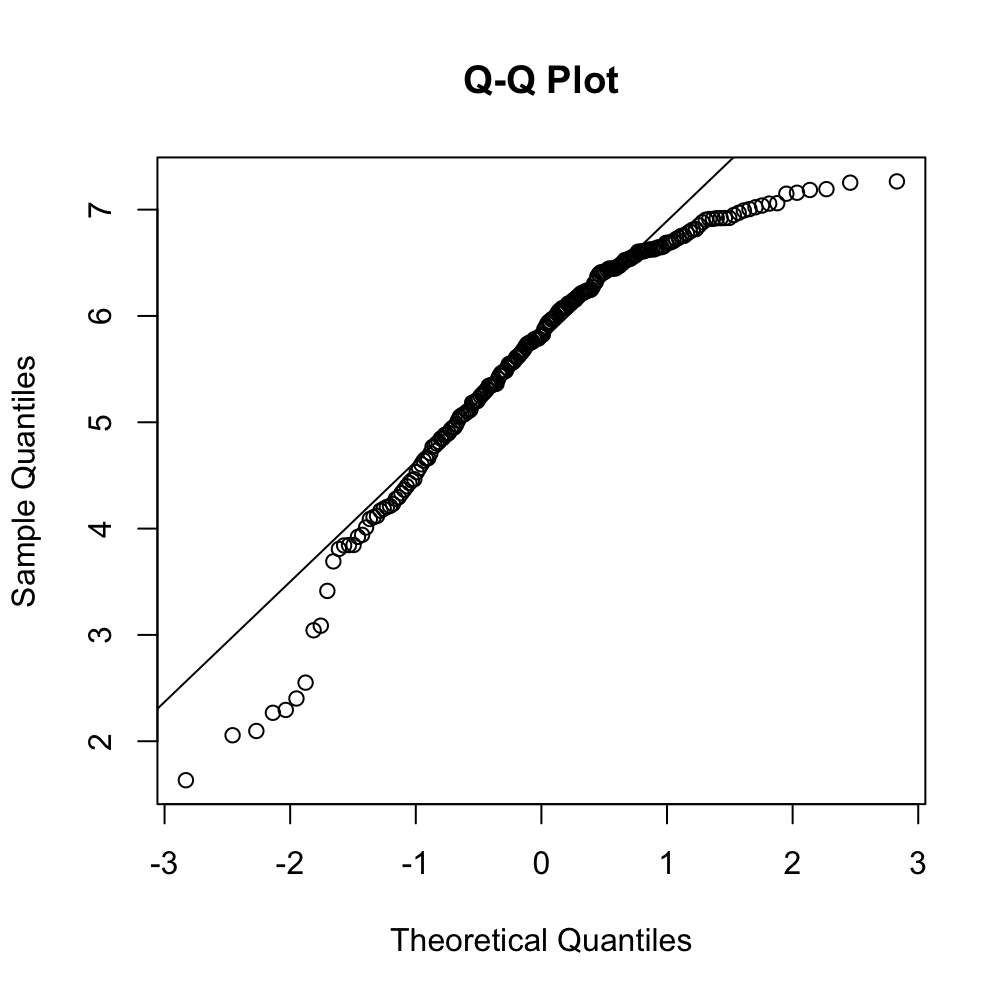


Figure S5 - Q-Q plot of Q0 data.
